# Posterior Fossa Astroblastoma: A Case Report

**DOI:** 10.7759/cureus.107357

**Published:** 2026-04-19

**Authors:** Ramazan Ozdemir, Gokhan Gurkan, Asli Kahraman, Ayse Karatas Demirciler

**Affiliations:** 1 Department of Neurosurgery, Izmir Katip Celebi University Atatürk Training and Research Hospital, İzmir, TUR; 2 Department of Pathology, Izmir Katip Celebi University Atatürk Training and Research Hospital, İzmir, TUR

**Keywords:** astroblastoma, case report, central nervous system tumors, cerebellum, gross total resection, posterior fossa

## Abstract

Astroblastoma is an extremely rare glial tumor that only rarely occurs in the posterior fossa. We report a case of posterior fossa astroblastoma in an adult patient to highlight its diagnostic challenges and management approach. A 38-year-old man presented with a two-week history of headaches and dizziness without focal neurological deficits. Cranial computed tomography (CT) and magnetic resonance imaging (MRI) revealed a well-defined 3.2 cm midline cerebellar mass containing both cystic and solid components, with a "cauliflower-like" enhancing solid portion and mild surrounding edema. The patient underwent a suboccipital craniotomy with complete microsurgical resection of the tumor, and he recovered well with no neurological deficits.

Histopathology confirmed astroblastoma, showing characteristic perivascular pseudorosettes and hyalinized blood vessels. Tumor cells were immunopositive for glial fibrillary acidic protein (GFAP), epithelial membrane antigen (EMA) in a dot-like pattern, and D2-40. No high-grade features (such as increased mitotic activity, microvascular proliferation, or significant nuclear atypia) were observed, consistent with a low-grade tumor. No adjuvant therapy was administered, and at three-year follow-up, the patient remains recurrence-free. Astroblastoma in the posterior fossa is exceptionally uncommon, posing a diagnostic challenge due to its nonspecific clinical and radiologic presentation. This case underscores that definitive diagnosis requires histopathological confirmation and that complete surgical resection can achieve favorable long-term outcomes. Long-term surveillance is recommended given the potential for late recurrence.

## Introduction

Astroblastoma was initially described by Bailey and Cushing in 1926 as a distinct glial tumor entity and was further characterized by Bailey and Bucy in 1930 [1]. In the World Health Organization (WHO) 2016 Classification of Tumors of the Central Nervous System, astroblastoma is listed among “other gliomas” and is not assigned a grade [2]. Astroblastomas are rare neuroepithelial central nervous system (CNS) tumors, accounting for 0.45-2.8% of all glial tumors, usually located in the cortex of the cerebral hemispheres [2-7]. Involvement of the midbrain, cerebellum, brainstem, and fourth ventricle has been rarely reported [5,8]. It has been reported to be twice as common in women as in men [8,9]. They typically show a bimodal age distribution, with the highest prevalence in children aged 5-10 years and young adults aged 21-30 years [7,10].

Radiologically, they usually appear as well-circumscribed, often mixed solid-cystic masses with contrast enhancement and minimal surrounding edema. Histopathologically, they are characterized by astroblastic pseudorosettes and vascular hyalinization [11], features that help distinguish them from ependymomas [8]. Typical immunohistochemical features include punctate staining with epithelial membrane antigen (EMA) and positive staining for glial fibrillary acidic protein (GFAP), S-100 protein, and vimentin [8]. In this article, we present a patient who underwent surgery for a posterior fossa mass, whose pathology was diagnosed as astroblastoma, and who has been followed for three years without recurrence.

## Case presentation

**Figure 1:**
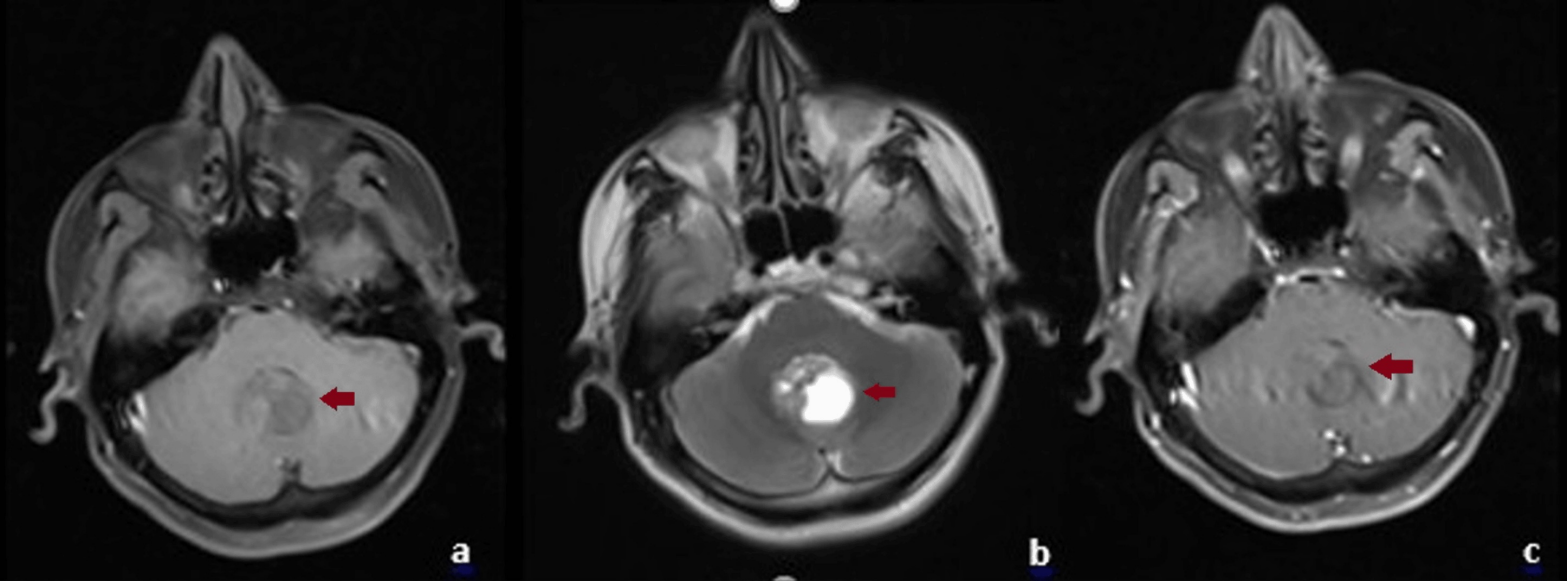
Axial magnetic resonance imaging (MRI) scans of the posterior fossa lesion (a) T1-weighted image showing a hypointense mass in the midline cerebellum. (b) T2-weighted image demonstrating a hyperintense signal with a cystic component. (c) Contrast-enhanced T1-weighted image revealing heterogeneous enhancement of the solid portion.

A 38-year-old male patient presented with a 15-day history of headache and dizziness. He denied nausea or vomiting. Detailed neurological examination revealed no signs of focal neurological deficit, including gait ataxia, cranial nerve palsy, or acute papilledema. There was no known comorbidity, prior surgery, or history of malignancy. He was a non-smoker. Cranial computed tomography (CT) showed a 3.2 cm midline cerebellar mass, with the solid component more prominent on the right and surrounded by edema. Magnetic resonance imaging (MRI) revealed a 3.2 cm midline posterior fossa lesion containing a cystic component, with contrast enhancement of the solid portion displaying a “cauliflower-like” appearance. On MRI sequences, the lesion appeared hypointense on T1-weighted images and hyperintense on T2-weighted images (Figure 1).

**Figure 2:**
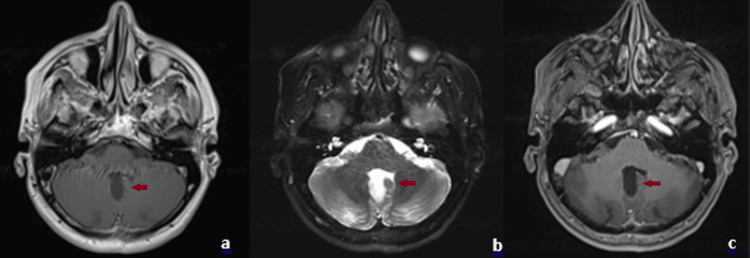
Postoperative axial magnetic resonance imaging (MRI) scans of the posterior fossa (a) T1-weighted image showing postoperative changes without residual tumor. (b) T2-weighted image demonstrating regression of perifocal edema. (c) Contrast-enhanced T1-weighted image revealing no pathological enhancement, confirming gross total resection.

The patient underwent a suboccipital craniotomy. Through a transvermian approach, the cystic component of the mass was accessed, and yellow cystic fluid was drained. The vascular solid component extending toward the fourth ventricle was completely excised under the surgical microscope. Postoperatively, the patient was admitted to the intensive care unit for monitoring. No motor or sensory deficits were noted. Cranial nerve examination and cerebellar function tests were normal. After 2 days in intensive care, he was transferred to the ward and discharged on postoperative day 6. Postoperative contrast-enhanced MRI showed no pathological enhancement (Figure 2).

Histopathological examination demonstrated classic features of astroblastoma, including perivascular pseudorosettes and hyalinized blood vessels. According to the 2021 WHO Classification of Central Nervous System Tumors, astroblastomas are characterized by immunopositivity for GFAP, EMA, and D2-40 [12], all of which were confirmed in this case (GFAP: positive; EMA: dot-like positive; D2-40: cytoplasmic and membranous positive). However, molecular testing for MN1 alteration-the diagnostic hallmark-could not be performed; therefore, the lesion was classified as “astroblastoma, NOS.”

In line with WHO 2021 recommendations, astroblastomas are not assigned a specific grade, but their low-grade versus high-grade morphological features should be reported. In our case, no high-grade histological features, such as increased mitotic activity, vascular proliferation, nuclear atypia, or architectural pattern loss, were observed. Immunohistochemistry further revealed S-100 positivity, while Olig2, Sox10, and p16 were negative; ATRX expression was preserved, and IDH1 was negative. The Ki-67 proliferation index was low (1-2%), and p53 expression was below 1%, indicating a low proliferative potential. Neurofilament staining showed no infiltrative growth pattern.

**Figure 3:**
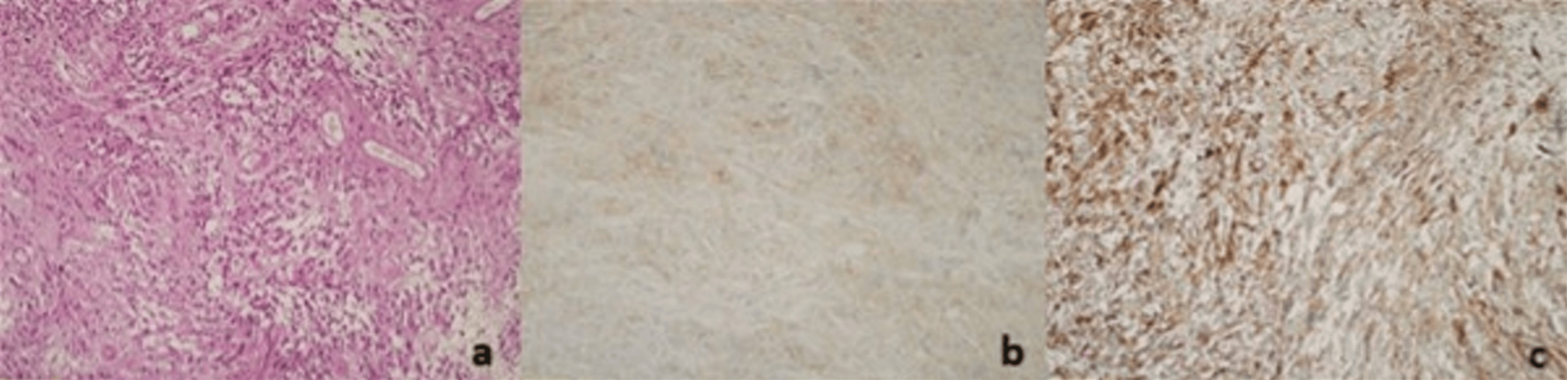
Histopathological and immunohistochemical findings of the tumor (a) Hematoxylin and eosin (H&E) staining showing perivascular pseudorosettes and hyalinized vessels (×10). (b) Immunohistochemical staining positive for D-240 (×10). (c) Immunohistochemical staining positive for glial fibrillary acidic protein (GFAP) (×10).

Taken together, these morphological and immunohistochemical findings support the diagnosis of astroblastoma, NOS, with low-grade histological characteristics. Following gross total resection, the tumor is expected to follow a clinical course similar to that of low-grade astroblastomas (Figure 3).

The pathology results, complete recovery, and postoperative imaging were discussed at a multidisciplinary neuro-oncology meeting. No adjuvant chemotherapy or radiotherapy was recommended. Follow-up MRI scans over three years showed no recurrence.

## Discussion

Astroblastoma is a rare CNS tumor, typically located in the supratentorial region [2-6]. However, it can rarely arise in the posterior fossa, including the cerebellum, brainstem, and fourth ventricle [13]. Although the typical age peaks are 5-10 and 21-30 years, cases can occur at any age [14]. Patients often present with nonspecific neurological symptoms related to increased intracranial pressure, either from obstructive hydrocephalus or direct brainstem compression [5]. Common symptoms include headache, nausea, and vomiting. MRI typically shows a well-demarcated, heterogeneous mass with mixed solid and cystic components. Lesions are usually hypointense on T1-weighted and hyperintense on T2-weighted images, with variable contrast enhancement and moderate diffusion restriction. Calcification is common in reported cases [13]. A “bubbly” appearance, due to multiple intratumoral cysts within the solid component, has been described [6]. Mild perifocal edema is usually present, and its extent does not correlate with tumor grade [4,15].

Histopathologically, astroblastomas show prominent vascularity, eosinophilic granular material, lymphocytic infiltration, rhabdoid cells, hyaline globules, and expression of both glial and epithelial markers [16]. Immunohistochemically, they are typically positive for GFAP, S-100 protein, and vimentin [5,17]. Methylation profiling is often required for definitive diagnosis, especially in challenging cases, to distinguish astroblastomas from other high-grade gliomas such as ependymomas or medulloblastomas. MN1 alterations are present in approximately 70% of cases [3].

Its rarity, overlapping imaging and histopathological features with those of other glial tumors, and the lack of a specific clinical presentation pose diagnostic challenges. The optimal treatment strategy is not clearly established. Gross total resection (GTR) remains the most significant prognostic factor for overall survival [11,18]. In malignant or recurrent cases, adjuvant radiotherapy-sometimes combined with chemotherapy-has been reported to improve local control, although outcomes remain variable [19].

In our case, the patient underwent gross total resection (GTR) and was followed with MRI every six months during the first postoperative year. No recurrence was observed over a three-year follow-up period. This case highlights the importance of distinguishing astroblastoma from ependymoma, given the therapeutic and prognostic differences between these entities. While the prognosis is generally favorable and recurrence is uncommon in well-differentiated astroblastomas, as in our case, tumors with malignant histopathological features-such as increased mitotic activity, vascular proliferation, nuclear atypia, and architectural pattern loss-may exhibit recurrence and even metastatic potential.

## Conclusions

Astroblastoma is an extremely rare CNS tumor, with posterior fossa involvement being exceptionally uncommon. Preoperative diagnosis is challenging due to its nonspecific radiologic and clinical features, and confirmation requires histopathological and immunohistochemical analysis. Gross total resection offers the best chance for long-term disease control. Long-term follow-up is essential, as recurrences may occur years after initial treatment. Further multi-institutional studies are needed to better define the role of adjuvant therapies and to establish standardized treatment protocols.
